# Brain–machine–brain interfaces as the foundation for the next generation of neuroprostheses

**DOI:** 10.1093/nsr/nwab206

**Published:** 2021-11-24

**Authors:** Miguel A L Nicolelis

**Affiliations:** Department of Neurobiology, Duke University, USA; International Institute of Neuroscience Edmond and Lily Safra, Brazil

**Figure 1.**
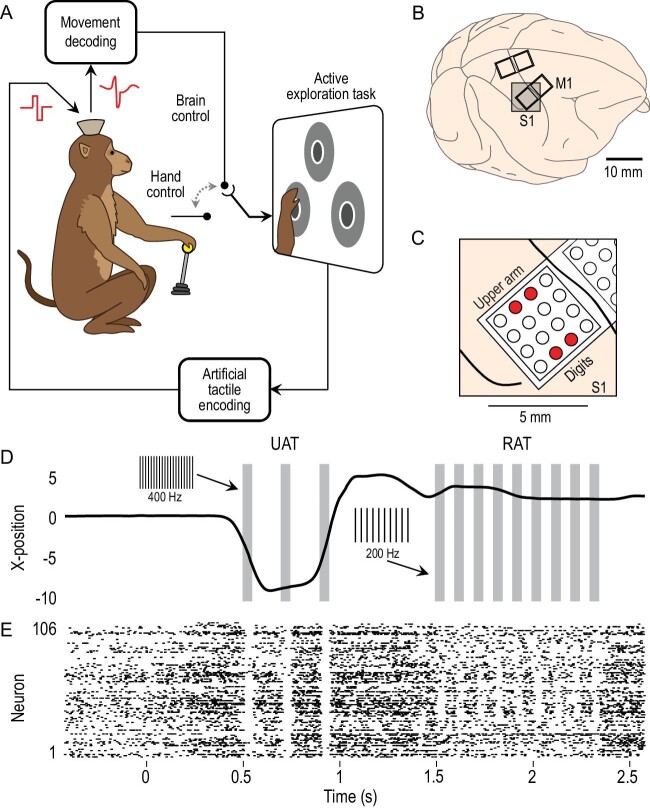
The brain–machine–brain interface. (A) While neuronal activity from M1 is used to extract movement control parameters, the S1 cortex is the main target of tactile feedback information. (B) Chronic implants of microwire electrodes targeted the M1 and S1 cortices. (C) Electrodes employed for intracortical microstimulation are highlighted in red. (D) Actuator movements when the subject explored the wrong targets but ultimately selected the correct one. Gray bars indicate stimulation patterns; insets indicate the intracortical microstimulation frequency; UAT: unrewarded artificial texture; RAT: rewarded AT. (E) Extracellular activity of an ensemble of M1 neurons sampled simultaneously during the trial shown in D. (Adapted with permission from Ref. [2]).

Back in 2011, my laboratory at the Duke University Center for Neuroengineering proposed a new variation on the already-classic paradigm known as brain–machine interfaces (BMIs) [1]. In a series of experiments [2], we showed that monkeys could not only learn to employ electrical signals from large neuronal ensembles (300–700 neurons) located in the primary motor cortex (M1) to control the movements of an avatar arm and hand—i.e. using a typical BMI paradigm—but that they could also interpret in real time, streams of electrical signals delivered directly to their primary somatosensory cortex (S1) via cortical microstimulation. This strategy was created to allow these monkeys to perform a tactile discrimination task, without any interference from their physical bodies. Thus, instead of using their real finger tips to perform such a task, this approach allowed our monkeys to use their brain activity to directly control the movements of ‘virtual finger tips’ to scan the texture of a series of virtual objects. Upon receiving, via cortical microstimulation, electrical signals directly into their S1 that were proportional to the virtual surface textures of these objects, the monkeys learned to quickly select the correct object that they needed to identify to receive a reward. Essentially, in this pioneering study and in a series of other experiments that followed it in the operation of what we named brain–machine–brain interfaces (BMBIs) (Fig. 1) [3,4], we demonstrated and introduced a paradigm that a decade later has the potential to guide a true revolution in the way we design and implement a new generation of neuroprostheses. Indeed, I envision that new technologies that at their core employ the BMBI concept may lead to a series of novel non-pharmacological therapies aimed at treating a series of neurological and psychiatric disorders that afflict hundreds of millions of people worldwide—the therapeutic management of which, even today, remains very challenging.

For this new generation of neuroprostheses to succeed, however, it will have to combine multiple approaches with the original BMBI paradigm introduced by my laboratory. Essentially, I believe that two key components will be essential for these devices to fulfill their goals. The first component will be responsible for continuously monitoring large-scale electrical brain activity in order to detect pathological signals related to the neurological condition of interest. For instance, in the case of untreatable epilepsy, either a non-invasive (e.g. EEG), semi-invasive (e.g. epidural recordings) or even invasive (e.g. intracranial electrode implants) method for detecting the initial signs of an upcoming epileptic seizure event will have to be implemented, just like in the study mentioned above [4]. Once a pathological brain activity pattern is identified and real-time algorithms predict that an upcoming critical event—a cortical seizure, for example—is very likely to happen, the second component of the neuroprosthesis will be activated. In my view, this second component will involve one or more methods for neurostimulation; either direct brain electrical stimulation via implants in different locations of the central nervous system, or the use of non-invasive methods, such as transcranial magnetic stimulation (TMS) or even transcranial direct or alternating current stimulation (tDCS and tACS). This approach is reminiscent of the type of brain-responsive neurostimulation approach already approved by the U.S. Food and Drug Administration (FDA) [5,6]. However, I envision further advances in the next few years that will go beyond this initial strategy for treating refractory chronic epilepsy and will apply to a variety of other brain diseases.

Successful experimental prototypes of this new generation of neuroprostheses have already been described in another large series of studies carried out in the USA and Brazil [7]. For example, in our BMBI, to monitor and block epileptic seizures in real time, we employed a semi-invasive method—electrical spinal cord stimulation (SCS)—to produce a robust reduction in the number and/or magnitude of seizures produced by a classic model of epilepsy [4]. The use of SCS in this latter case was inspired by our previous discovery that high-frequency SCS, delivered at the cervicothoracic junction, could be successfully employed to alleviate untreatable ‘gait freezing’ and other cardinal symptoms of animal models of Parkinson's disease [7], a finding that has been recently reproduced in Parkinsonian patients by many independent groups worldwide (for a review see [8]). Curiously, SCS was seen as a potential choice for treating Parkinsonian gait freezing because of one prior experimental observation and one new theory of brain disorders. The observation was that electrical stimulation of the trigeminal nerve could induce anti-seizure effects compared to what is obtained by vagus nerve stimulation [9]. Since we demonstrated that in animal models of Parkinson's disease the entire motor system seems to become engaged in a pattern of neuronal hypersynchronization that resembles focal epilepsy, we postulated that proper electrical stimulation of the CNS should disrupt these ‘Parkinsonian seizures’ and lead to significant amelioration of gait freezing in both animals and patients. This prediction was fully confirmed experimentally as mentioned above.

Based on these and a series of other studies involving a number of transgenic mice models of neurologic and psychiatric disorders [10], I have proposed that most neurological and psychiatric disorders may share a common physiopathological pathway, which generates their clinical signs and symptoms in patients [10]. That common pathway would involve, for each specific disease no matter its original cause (genetic, metabolic, immunological, traumatic etc.), the recruiting of large populations of neurons of a particular brain circuit into a state of hyperexcitability like the one associated with focal epilepsy. According to this view, diseases such as Parkinson's, Alzheimer's, migraines, bipolar disorder, schizophrenia, obsessive compulsive disorder, depression, anxiety, essential tremor, autism, eating disorders and many other brain disorders could manifest themselves clinically as a result of focal epileptic seizures taking part in specific brain circuits involving both cortical and subcortical structures [10]. If this theory proves correct, the coupling of BMBIs and a variety of neurostimulation approaches, like electrical or TMS-based stimulation of the dorsal columns of the spinal cord, could lead to the emergence of a new generation of neuroprostheses capable of providing non-pharmacological and on-demand therapy for a large number of patients suffering from some of the most devastating neurological and psychiatric disorders that today affect hundreds of millions of people worldwide.

***Conflict of interest statement*.** None declared.
